# PCNA Ser46-Leu47 residues are crucial in preserving genomic integrity

**DOI:** 10.1371/journal.pone.0285337

**Published:** 2023-05-19

**Authors:** Sangin Kim, Yeongjae Kim, Youyoung Kim, Suhyeon Yoon, Kyoo-young Lee, Yoonsung Lee, Sukhyun Kang, Kyungjae Myung, Chang-Kyu Oh

**Affiliations:** 1 Institute for Basic Science, Center for Genomic Integrity, Ulsan, Korea; 2 Department of Biological Sciences, Ulsan National Institute of Science and Technology, College of Information-Bio Convergence Engineering, Ulsan, Korea; 3 National Institute of Allergy and Infectious Diseases, National Institutes of Health, Integrated Data Sciences Section, Research Technologies Branch, Bethesda, MD, United States of America; 4 Department of Biochemistry, College of Medicine, Hallym University, Chuncheon, Gangwon-do, Korea; 5 Clinical Research Institute, Kyung Hee University Hospital at Gangdong, College of Medicine, Kyung Hee University, Seoul, Korea; 6 Ulsan National Institute of Science and Technology, Department of Biomedical Engineering, College of Information-Bio Convergence Engineering, Ulsan, Korea; 7 Department of Biochemistry, Pusan National University, School of Medicine, Yangsan, Korea; Georgetown University, UNITED STATES

## Abstract

Proliferating cell nuclear antigen (PCNA) is a maestro of DNA replication. PCNA forms a homotrimer and interacts with various proteins, such as DNA polymerases, DNA ligase I (LIG1), and flap endonuclease 1 (FEN1) for faithful DNA replication. Here, we identify the crucial role of Ser46-Leu47 residues of PCNA in maintaining genomic integrity using *in vitro*, and cell-based assays and structural prediction. The predicted PCNA^ΔSL47^ structure shows the potential distortion of the central loop and reduced hydrophobicity. PCNA^ΔSL47^ shows a defective interaction with PCNA^WT^ leading to defects in homo-trimerization *in vitro*. PCNA^ΔSL47^ is defective in the FEN1 and LIG1 interaction. PCNA ubiquitination and DNA-RNA hybrid processing are defective in PCNA^ΔSL47^-expressing cells. Accordingly, PCNA^ΔSL47^-expressing cells exhibit an increased number of single-stranded DNA gaps and higher levels of γH2AX, and sensitivity to DNA-damaging agents, highlighting the importance of PCNA Ser46-Leu47 residues in maintaining genomic integrity.

## Introduction

The eukaryotic sliding clamp, proliferating cell nuclear antigen (PCNA), is a master coordinator of DNA replication fork-associated processes. PCNA plays a crucial role in DNA replication, DNA damage bypass, DNA repair, chromatin remodeling, cohesion establishment, and sister chromatid exchange [1–4]. PCNA interacts with various proteins by providing a binding platform [1,5,6]. During DNA replication, PCNA is loaded onto DNA by replication factor C (RFC) and unloaded by the ATAD5-RFC-like complex (RLC) after DNA synthesis [7–9].

The three PCNA monomers form a homo-trimeric complex with a ring-shaped structure for faithful DNA replication and DNA synthesis [10]. Defects in PCNA trimerization impair DNA replication in cells [11]. ATP-bound RFC binds to and opens a PCNA homo-trimeric ring to load PCNA onto the DNA [12–14]. In yeast, *pol30* mutations at E113G, L151S, or G178S, which cannot form trimeric complexes properly, inhibit translesion synthesis (TLS) and prevent mutagenesis, but not RAD6/18-dependent PCNA mono-ubiquitination [15,16]. The PCNA homotrimer has structural features with two parts having different putative roles [1]. The front part interacts with various partner proteins, such as DNA polymerase δ, RFC, flap endonuclease 1 (FEN1), DNA ligase I (LIG1), cyclin-dependent kinase 2 (CDK2), cyclin D, and other DNA metabolism proteins [1,17]. PCNA ubiquitination at the K164 residue by RAD18 occurs at the back part of PCNA, and Y-family TLS polymerase polη is known to recognize the ubiquitin moiety of the ubiquitinated-PCNA (Ub-PCNA) [18,19]. Therefore, the front part ensures the processivity of DNA replication, while the back plays a role linked to the Ub-PCNA and TLS pathways.

The central loop (^41^DSSH^44^) and inter-domain connecting loop (IDCL) (^121^LDVEQLGIPEQE^132^) of PCNA are evolutionarily conserved and located on the front part. These residues play crucial roles in the binding of multiple PCNA-associated proteins. Both the center loop and IDCL do not contain a positively charged arginine (R) or lysine (K) residue and therefore serve as the hydrophobic core. [20–24]. Notably, the IDCL is crucial for PCNA trimerization [10,17,25]. PCNA Ser43-His44-Val45 residues are located in the βC_1_–βD_1_ loop containing the central loop, and this loop is known to be important for PCNA loading onto DNA by RFC *in vitro* [17]. Double mutations of yeast *pol30* (human PCNA) at DD41, 42AA cause defects in Pol δ stimulation and Pol ε-mediated DNA synthesis, resulting in growth defects [26]. In humans, the PCNA D41A mutation causes severe defects in both the stimulation of RFC ATPase activity and the processivity of Pol δ. The Val45 residue of PCNA is important for the formation of a hydrophobic surface interacting with p21 [27,28]. In addition, analyses of PCNA mutations have revealed that the major RFC interaction sites are the central loop (Asp41-His44) and the carboxyl-terminal tail (Lys254-Glu256), both of which protrude on one face called the C-side [17,23,27,29]. Importantly, the hypomorphic PCNA S228I mutation underlies a human DNA repair disorder associated with a neurodegenerative phenotype. The PCNA S228I mutation impairs FEN1 and LIG1 interactions and substantially reduces in UV survival and RNA synthesis recovery [30].

Herein, we identified the role of conserved and crucial PCNA residues Ser46-Leu47 in PCNA trimerization and ubiquitination using structure prediction, *in vitro*, and cell-based assays. Deletion of two amino acids, Ser46-Leu47 (PCNA^ΔSL47^), structurally distorts the central loop region and reduces its hydrophobicity. The PCNA^ΔSL47^ mutant-expressing cells are defective in interaction with FEN1 and LIG1, and PCNA ubiquitination. PCNA^ΔSL47^-expressing cells show an increased level of cellular DNA-RNA hybrids and γH2AX. Accordingly, PCNA^ΔSL47^-expressing cells are sensitive to genotoxic stress, which highlights the importance of Ser46-Leu47 residues of PCNA in preserving genomic integrity.

## Results

### The structural importance of PCNA Ser46 and Leu47 residues in forming a central loop and hydrophobic core formation

**Fig 1:**
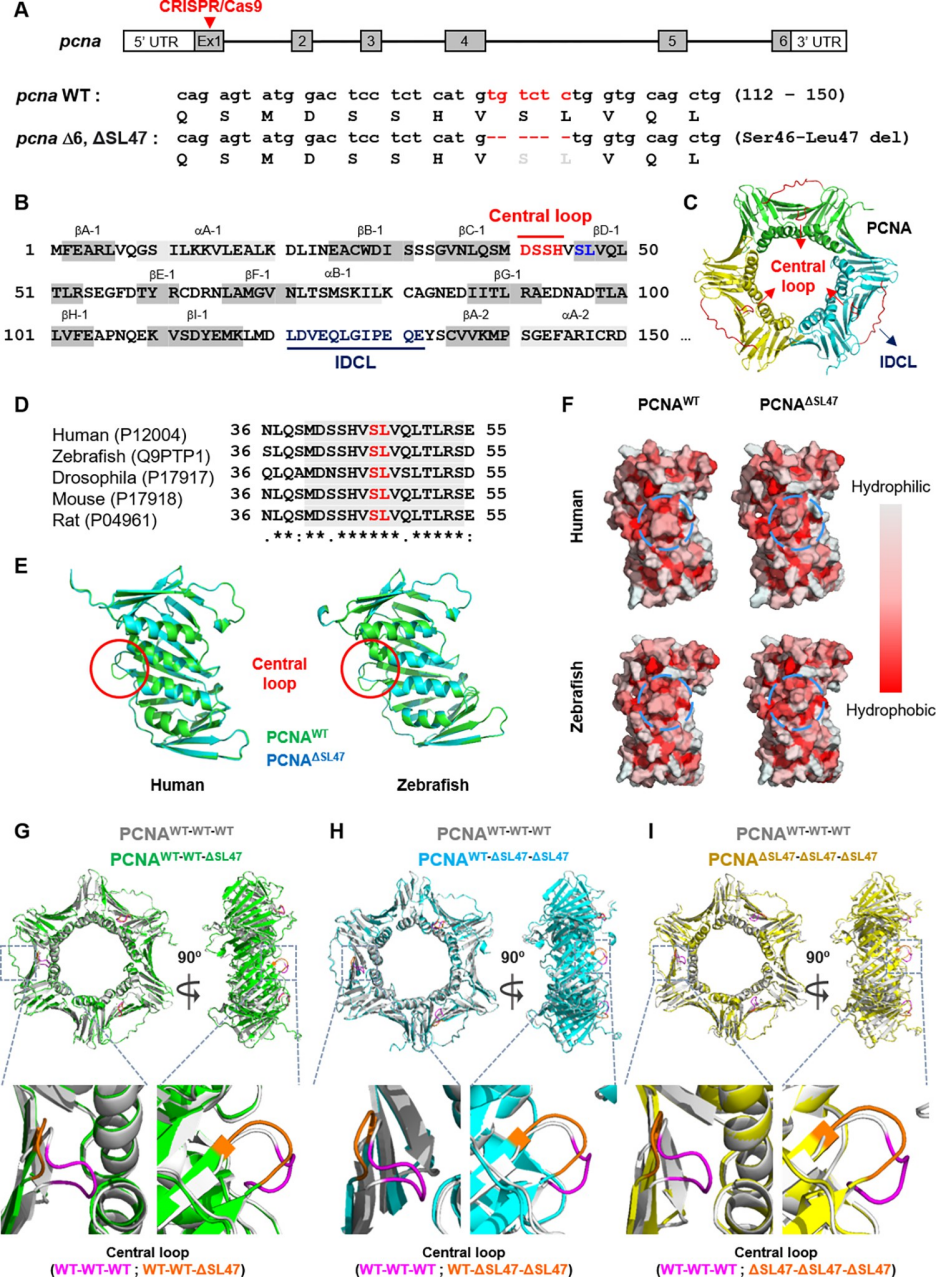
The structural importance of PCNA Ser46, and Leu47 residues in a central loop and hydrophobic core formation. (A) (Upper) Diagram indicates all the available coding exons (gray bar) in the *pcna* gene and the targeted exon region is indicated with red arrowheads. (Bottom) DNA sequence (112–150) of zebrafish *pcna* and the sequences of sgRNA target site between WT and *pcna* in-frame mutant (ΔSer46-Leu47, ΔSL47) in red. Putative translated amino acid sequences are indicated below the DNA sequences. Hyphens indicate deleted sequence of *pcna* ΔSL47 mutant. (B) Amino acid sequence and the secondary structure of human PCNA (P12004) are shown. α-helices and β-strands are indicated above the amino acid sequence. Ser46 and Leu47 residues are colored in blue. (C) Human PCNA structure containing central loop and inter-domain connector loop (IDCL) regions marked with red. (D) Conserved residues in PCNA. (E) Predicted monomer structures of PCNA^WT^ (Green) or PCNA^ΔSL47^ (Cyan) in both human and zebrafish. The red circle indicates the central loop region. (F) Hydrophobicity of PCNA^WT^ and PCNA^ΔSL47^ monomers around PCNA central loop region. The closer the surface color is to red, the more hydrophobic it is. Hydrophobicity is analyzed in both human and zebrafish. The blue circle indicates the Ser46-Leu47 residues. (G-I) Predicted trimerized PCNA structure analyzed with corresponding combinations in human. PCNA WT-WT-WT homotrimer (Gray) are structurally aligned with either WT-WT-ΔSL47 (Green) (G), WT- ΔSL47-ΔSL47 (Cyan) (H), ΔSL47-ΔSL47-ΔSL47 (Yellow) (I). Both the front- and side- views are displayed (Top). The central loop regions are colored as indicated (Bottom).

We previously targeted exon 1 of the *pcna* gene using the CRISPR/Cas9 system and acquired several zebrafish homozygous mutants [31], and found that targeted and deleted DNA sequences were mainly associated with and translated to, conserved Ser46-Leu47 amino acids (Fig 1A) [31]. Considering PCNA is a key protein in DNA replication and repair [2], we wanted to elucidate the molecular mechanisms underlying the phenotypes in *pcna*^ΔSL47/ΔSL47^ mutants. PCNA Ser46-Leu47 residues were adjacent to the central loop and conserved across species (Fig 1B–1D). To gain structural insight into Ser46-Leu47 residues in a central loop formation, we predicted the PCNA monomer structures in both *Homo sapiens* (hereafter, human) and *Danio rerio* (hereafter, zebrafish) using wild-type PCNA (PCNA^WT^) and PCNA^ΔSL47^ using Alphafold2 (Fig 1E and 1F). The highest-ranked prediction model of the PCNA^ΔSL47^ monomer was comparable to that of the PCNA^WT^ monomer, except for the central loop formation. Interestingly, PCNA^ΔSL47^ did not have a predominant protrusion compared with PCNA^WT^ (Fig 1E). Because this dent structure exposes the hydrophilic βD-1 structure, the hydrophobicity within the hydrophobic core region of the central loop was checked. We found that PCNA^ΔSL47^ had dramatically diminished hydrophobicity compared to the PCNA^WT^ protein (Fig 1F). Consistent with the conserved motif of this region across species (Fig 1D), structural central loop distortion and the altered hydrophobicity between PCNA^WT^ and PCNA^ΔSL47^ were also conserved in both humans and zebrafish (Fig 1E and 1F).

To gain insight into whether this altered central loop formation and hydrophobicity affect PCNA trimerization, we predicted trimerized PCNA structures with the PCNA^WT^ and PCNA^ΔSL47^ mutants in various combinations as follows: (1) WT-WT-WT; (2) WT-WT-ΔSL47; (3) WT-ΔSL47-ΔSL47; (4) ΔSL47-ΔSL47-ΔSL47. Consistent with the monomer prediction results, when PCNA^ΔSL47^ mutants were included during PCNA trimerization, the exposed surface and length of the PCNA central loop region were reduced compared to the PCNA^WT^ homotrimer (Fig 1G–1I). Consistent with the conserved structural phenotypes of PCNA^WT^ and PCNA^ΔSL47^ monomers, we also observed similarly distorted central loop formation in zebrafish (S1A-S1C Fig). Taken together, the deletion of PCNA Ser46-Leu47 residues might distort PCNA, which could be accompanied by changes in the hydrophobicity of the central loop region in zebrafish and humans.

### Purified PCNA^ΔSL47^ mutant protein forms non-trimeric heterogenous multimers *in vitro*

**Fig 2:**
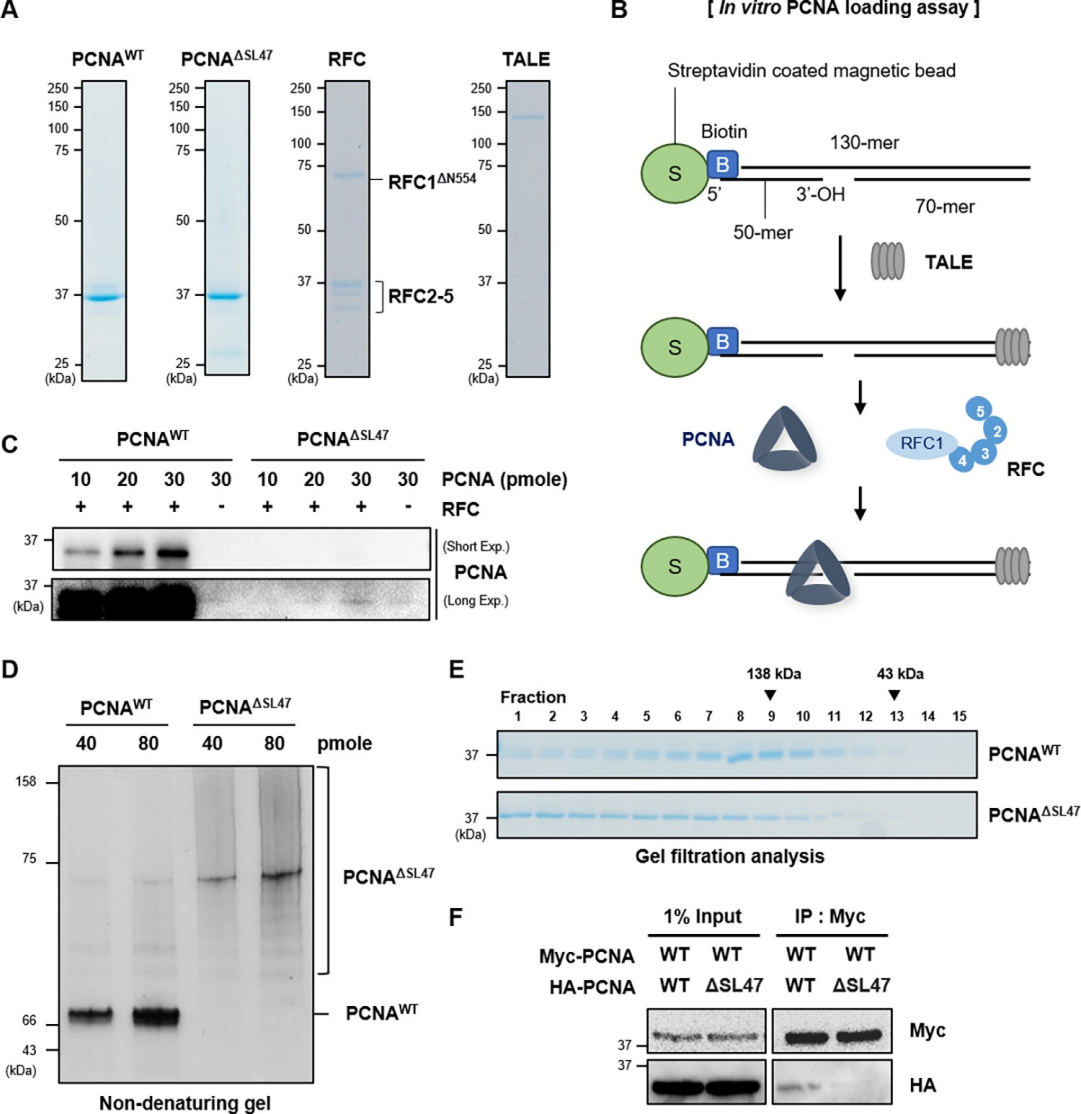
Purified PCNA^ΔSL47^ protein forms non-trimeric heterogenous multimers *in vitro*. (A) Coomassie-stained SDS-PAGE of purified PCNA^WT^, PCNA^ΔSL47^, RFC, and TALE proteins. (B) Schematic diagram of PCNA loading assay. 5’-biotinylated DNA substrate was attached to the streptavidin-coated magnetic beads. The free end of DNA was blocked by the TALE protein. PCNA was loaded to the substrate DNA by purified RFC. (C) DNA-loaded PCNA is analyzed by immunoblotting. (D) The indicated amount of purified PCNA^WT^ or PCNA^ΔSL47^ was separated on 10% non-denaturing gel and stained with Coomassie blue. (E) Gel filtration analysis with purified PCNA^WT^ or PCNA^ΔSL47^ mutant was performed and stained with Coomassie blue. (F) Myc- or HA-tagged PCNA constructs were expressed in HEK293T cells and subjected to immunoprecipitation (IP) with anti-Myc affinity beads. Immunoprecipitated proteins were eluted, resolved by SDS-PAGE, and immunoblotted using the indicated antibodies.

To verify whether PCNA^ΔSL47^ mutant proteins carry out functional PCNA activities, such as loading onto DNA by RFC and trimer formation, we purified recombinant human PCNA^WT^ and PCNA^ΔSL47^ mutant proteins from *E*. *coli* (Fig 2A). Since the Ser46-Leu47 residues are structurally adjacent to the central loop and could affect DNA loading, we investigated whether the PCNA^ΔSL47^ mutant protein can be loaded onto DNA. We performed an *in vitro* PCNA loading assay, as previously described (Fig 2B) [7]. Although PCNA^WT^ proteins were loaded onto DNA in a dose-dependent manner by RFC, PCNA^ΔSL47^ mutant proteins showed a significant defect in loading onto DNA by RFC (Fig 2C).

Since the PCNA^ΔSL47^ mutant protein was barely loaded onto DNA by RFC *in vitro* presumably due to structural changes in the central loop (Fig 1), the PCNA^ΔSL47^ mutant protein was then investigated for its ability to form a trimer. Using a non-denaturing gel, we compared the native states of PCNA^WT^ and PCNA^ΔSL47^ proteins (Fig 2D). Due to the faster migration speed of ring-shaped structures in non-denaturing gels compared to linear proteins, the PCNA^WT^ homotrimer band appeared at a lower position (66 kDa) than its molecular weight (90 kDa). In the case of PCNA^ΔSL47^, we observed a major band (75 kDa) appearing at a larger size than PCNA^WT^. PCNA^ΔSL47^ protein had a lower migration speed than PCNA^WT^, despite the fact that amino acids are deleted compared to PCNA^WT^ homo-trimer. Furthermore, unlike with PCNA^WT^, abundant protein bands were visible at sizes above the major band (75 kDa) in PCNA^ΔSL47^ (Fig 2D). This data suggests that PCNA^ΔSL47^ has a slower and more diverse range of migration speeds. This pattern typically appears when proteins aggregate. Therefore, it is likely that PCNA^ΔSL47^ protein does not form a regular ring-shaped structure *in vitro* or forms higher-order complexes larger than a homotrimer, and is prone to aggregation.

To test PCNA^ΔSL47^ mutant proteins form higher order complexes (or aggregations), we performed gel filtration analysis. PCNA^WT^ proteins eluted mostly at ~120 kDa, similar to the PCNA homotrimer molecular mass (Lane 7–10) (Fig 2E), consistent with a previous report [32]. In contrast, PCNA^ΔSL47^ mutant proteins eluted in a broad range of molecular masses (Lane 1–6), and the expected sizes were higher than that of the PCNA^WT^ protein. PCNA^ΔSL47^ protein was eluted more quickly compared to PCNA^WT^, indicating that PCNA^ΔSL47^ forms the higher order of oligomers or aggregation (Fig 2E).

Finally, we investigated whether PCNA^ΔSL47^ mutant monomers form a trimer with PCNA^WT^ in cells. To test this hypothesis, we performed an immunoprecipitation assay using anti-c-Myc-agarose while co-overexpressing Myc-tagged PCNA^WT^ with *hemagglutinin* (HA)-tagged PCNA^WT^ or PCNA^ΔSL47^. Interestingly, Myc-tagged PCNA^WT^ interacted with HA-tagged PCNA^WT^ but not with the HA-tagged PCNA^ΔSL47^ mutant (Fig 2F). Taken together, these data suggest that the PCNA^ΔSL47^ mutant protein is defective in PCNA trimerization and forms a higher order of oligomers or aggregation.

### PCNA^ΔSL47^ mutant interacts with RFC subunits but fails to interact with FEN1 and LIG1

**Fig 3:**
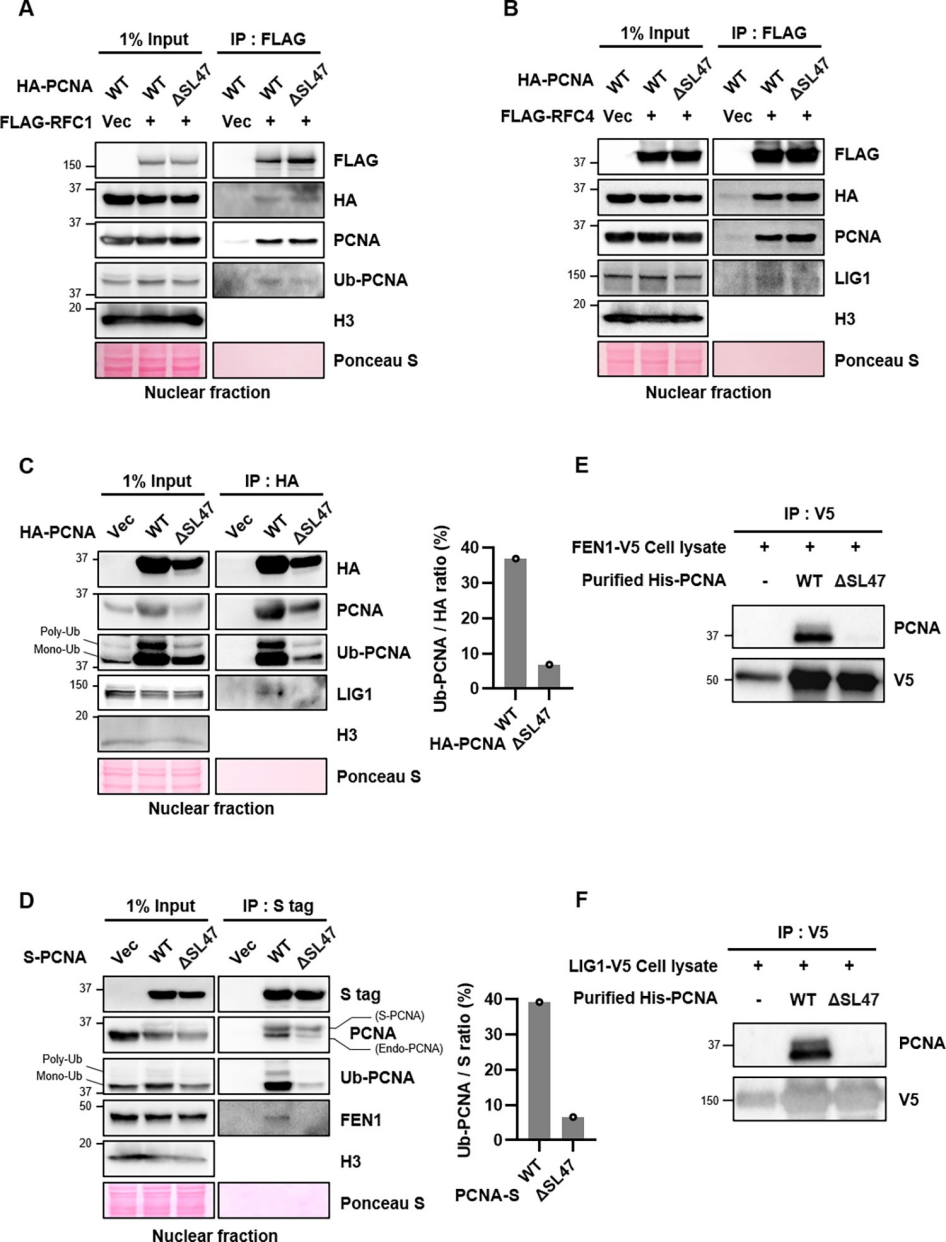
PCNA^ΔSL47^ mutant interacts with RFC subunits but fails to interact with FEN1 and LIG1. (A-F) Immunoprecipitated proteins are eluted, resolved by SDS-PAGE, and immunoblotted using the indicated antibodies. (A-D) After transfection in HEK293T cells as indicated, nuclear extracts were prepared for immunoprecipitation (IP) with an anti-FLAG affinity (A and B) or anti-HA affinity (C), or anti-S affinity bead (D). (C and D) The intensity ratio of either Ub-PCNA and HA (C) or Ub-PCNA and S (D) was quantified and plotted. (E and F) After transfection with either V5-tagged FEN1 or LIG1 in HEK293T cells for 48 h, whole-cell lysates were prepared and incubated with purified PCNA protein for 1 h.

We previously observed that the PCNA^ΔSL47^ mutant can be loaded onto chromatin [31]. However, the purified PCNA^ΔSL47^ mutant protein could not be loaded onto DNA, and it failed to form a trimer *in vitro* (Fig 2C–2F). To verify whether the PCNA^ΔSL47^ mutant interacts with RFC subunits, we performed an immunoprecipitation assay in RFC1- or RFC4-expressing cells (Fig 3A and 3B). Interestingly, RFC1 interacted with both PCNA^WT^ and PCNA^ΔSL47^ mutant proteins (Fig 3A). Since PCNA interacts with RFC1 via multiple binding sites [14], we suspect that both PCNA^WT^ and the PCNA^ΔSL47^ mutant can interact with RFC1. In addition to RFC1, RFC4 also interacted with both PCNA^WT^ and PCNA^ΔSL47^ mutants (Fig 3B).

We then investigated whether the PCNA^ΔSL47^ mutant affects its interaction with FEN1 or LIG1. To test this, we overexpressed HA-tagged PCNA^WT^ or PCNA^ΔSL47^ and performed an immunoprecipitation assay. We observed that the HA-PCNA^ΔSL47^ mutant failed to interact with LIG1, whereas HA-PCNA^WT^ interacted with endogenous LIG1 (Fig 3C). Next, we tested whether endogenous FEN1 interacts with PCNA^WT^ or PCNA^ΔSL47^ by overexpressing S-tagged PCNA^WT^ or PCNA^ΔSL47^. Interestingly, the S-PCNA^ΔSL47^ mutant failed to interact with FEN1, whereas S-PCNA^WT^ interacted with endogenous FEN1 (Fig 3D). We suspect that the PCNA central loop region is structurally distorted, and its hydrophobicity exposure to FEN1 or LIG1 is impaired in PCNA^ΔSL47^ (Fig 1), which is observed in the PCNA^SHV43AAA^ mutant [17]. This may result in defects in FEN1 and LIG1 interactions. To further confirm this, we incubated either FEN1- or LIG1- overexpressed cell lysates with purified His-tagged PCNA^WT^ or PCNA^ΔSL47^ mutant protein, respectively (Fig 3E and 3F). By performing an immunoprecipitation assay using an anti-V5 antibody, we observed that both FEN1 and LIG1 interacted with PCNA^WT^ (Fig 3E and 3F). Neither FEN1 nor LIG1 interacted with PCNA^ΔSL47^ mutant protein. We concluded that PCNA Ser46 and Leu47 residues are not required for interaction with RFC1 and/or RFC4, but are required for interaction with FEN1 and LIG1.

### PCNA mono-ubiquitination is defective in PCNA^ΔSL47^-expressing cells

**Fig 4:**
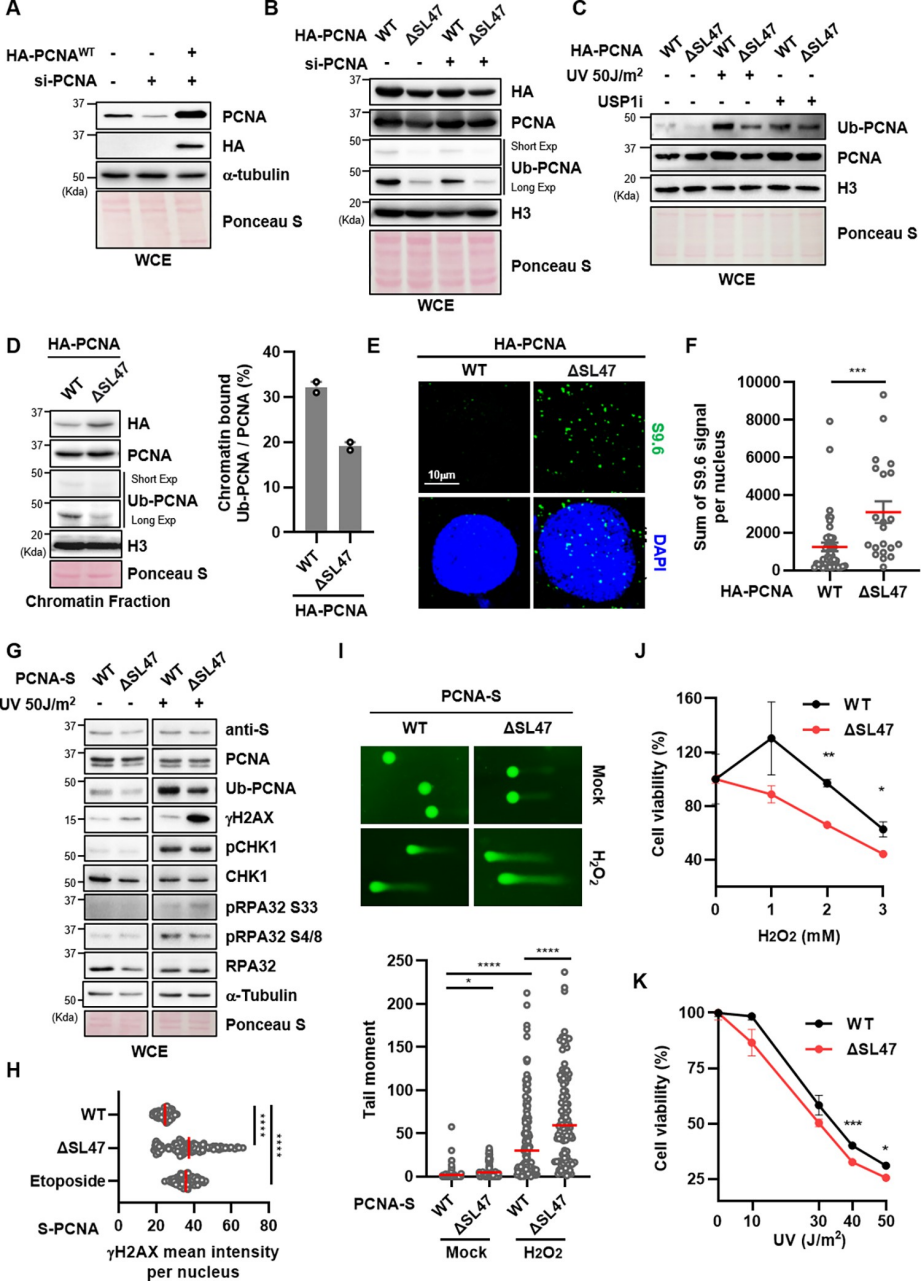
PCNA Ser46 and Leu47 amino acids are important for genomic stability in cells. (A and B) HEK293T cells were transfected with a combination of cDNA expressing HA-PCNA^WT^ (A and B) or HA-PCNA^ΔSL47^ (B) and *PCNA* siRNA targeting 3’UTR for 48 h. Whole-cell extracts were prepared for immunoblotting. (C) After cDNA transfection as indicated, cells were irradiated with 50 J/m^2^ UV and recovered for 6 h or treated for 6 h with 30 μM USP1 inhibitor (ML323). Whole-cell extracts were isolated and immunoblotted. (D) HEK293T cells were transfected with cDNA expressing HA-PCNA^WT^ or HA-PCNA^ΔSL47^ for 48 h and chromatin-bound fractions were prepared for immunoblotting. The intensity ratio of Ub-PCNA and PCNA was quantified and plotted. (E and F) HeLa cells were transfected with cDNA expressing HA-tagged PCNA^WT^ or PCNA^ΔSL47^ for 48 h, and immunostained with the anti-S9.6 antibody. (E) Representative images of S9.6 immunostaining. (F) The sum intensity of S9.6 foci was quantified and plotted. (G) After cDNA transfection as indicated in HEK293T cells, cells were irradiated with 50 J/m^2^ UV and recovered for 6 h. Whole-cell extracts were prepared for immunoblotting. (H) HeLa cells were transfected with cDNA expressing S-tagged PCNA^WT^ or PCNA^ΔSL47^ for 48 h, and immunostained with the anti-γH2AX antibody. 10 μM of etoposide was treated for 5 h as a positive control. The mean intensity of γH2AX staining was quantified and plotted. (I) (Upper) Representative images of alkaline COMET assay. U2OS cells were transfected with indicated cDNAs for 48 h, treated with 0.1 mM H_2_O_2_ for 1 h, and collected for an alkaline COMET assay. (Below) The tail moments were quantified and plotted. (J and K) U2OS cells were transfected as indicated for 48 h, and treated with H_2_O_2_ for 4 h with MTT reagents (J) or irradiated with UV as indicated (K). Error bars represent the standard deviation of the mean. (J) Cell survival was measured by quantification of MTT formazan. (K) Cells were recovered for 48 h after UV-irradiation, and cells were subjected to CellTiter-Glo® assay. (E, F, H, I, J, and K) Three independent experiments were performed, and one representative result is displayed. The red bar indicates the mean value. Statistical analysis: two-tailed unpaired Student’s *t*-test, **** *p* < 0.0001, *** *p* < 0.001, ** *p* <0.01, * *p* < 0.05.

When DNA replication encounters lesions, PCNA is ubiquitinated at the K164 residue for damage bypass [33]. Therefore, we examined whether PCNA^ΔSL47^ mutants, which have defects in trimerization, FEN1 and/or LIG1 interaction, have an impact on PCNA ubiquitination. To assess the ubiquitination of PCNA^ΔSL47^ with minimal effect by endogenous PCNA in cells, we depleted endogenous PCNA using siRNA targeting the 3’-untranslated region (UTR) and expressed either HA-tagged PCNA^WT^ or PCNA^ΔSL47^ in the cell. The siRNA-mediated knockdown efficiency and expression of HA-PCNA^WT^ and HA-PCNA^ΔSL47^ were confirmed by immunoblotting (Fig 4A and 4B). Interestingly, basal PCNA ubiquitination was defective in PCNA^ΔSL47^-expressing cells (Fig 4B). Moreover, PCNA^ΔSL47^ diminished the interaction with Ub-PCNA compared with PCNA^WT^ (Fig 3C and 3D). To further confirm this, we irradiated cells with UV to induce RAD18-mediated PCNA mono-ubiquitination [34–36] or treated a ubiquitin-specific protease 1 (USP1) inhibitor (hereafter USP1i, ML323) to block Ub-PCNA deubiquitination by the USP1-USP1-associated factor 1 (UAF1) complex [37]. Both UV irradiation and USP1i treatment increased Ub-PCNA levels (Fig 4C). Consistent with the unperturbed conditions (Fig 4B), we observed lower Ub-PCNA levels in PCNA^ΔSL47^-expressing cells than in PCNA^WT^-expressing cells under UV- and/or USP1i-treated conditions (Fig 4C). Finally, since PCNA should be loaded onto chromatin for its ubiquitination *in vitro* [7,38], we investigated the chromatin-loaded Ub-PCNA/PCNA ratio. Consistent with our previous result [31], PCNA^ΔSL47^ was loaded onto chromatin similar to PCNA^WT^ in cells. However, the ratio of Ub-PCNA/PCNA in the chromatin fraction was significantly diminished (Fig 4D). Taken together, our data suggest that PCNA Ser46-Leu47 residues are crucial for PCNA ubiquitination.

### PCNA Ser46 and Leu47 amino acids are important for genomic stability in cells

We next investigated whether the PCNA^ΔSL47^ mutant affects genomic integrity in cells using various assays. Recent studies have shown that PCNA unloading, PCNA ubiquitination, and FEN1 are associated with DNA-RNA hybrids and R-loop regulation [39–42]. Since PCNA^ΔSL47^ showed a PCNA ubiquitination defect (Figs 3C, 3D and 4B–4D), similar to RAD18-deficient cells [40], we next assessed whether the PCNA^ΔSL47^ mutant affects cellular DNA-RNA hybrid levels. By performing immunostaining with the S9.6 antibody with metaphase spread, we measured the DNA-RNA hybrid levels in the nucleus (Fig 4E). Interestingly, PCNA^ΔSL47^ mutant-expressing cells showed increased cellular DNA-RNA hybrid levels in the nucleus compared to PCNA^WT^-expressing cells (Fig 4E and 4F).

Next, we checked the γH2AX level by performing both immunoblotting and immunostaining. As a result, we could observe increased γH2AX level in PCNA^ΔSL47^-expressing cells, compared to PCNA^WT^-expressing cells in both unperturbed conditions (Fig 4G and 4H), and UV-irradiated conditions (Fig 4G). As a positive control for γH2AX staining, cells were treated with 10 μM etoposide for 5 h (Fig 4H). In addition, we checked other DNA damage response markers such as pRPA32 S33, pRPA32 S4/8, and pCHK1 [43], and observed an increase in phosphorylation of RPA32 S33 (pRPA32 S33) in PCNA^ΔSL47^-expressing cells compared to PCNA^WT^-expressing cells under UV-irradiated condition. However, we could not observe changes in other DNA damage response markers such as pCHK1, and pRPA32 S4/8 (Fig 4G).

Accordingly, we performed the alkaline comet assay (single-cell gel electrophoresis) to measure the single-stranded DNA gaps [44], and the tail moment was increased in PCNA^ΔSL47^-expressing cells in H_2_O_2_-treated condition (Fig 4I). We also checked the cell survival upon either H_2_O_2_ treatment or UV irradiation. As a result, we observed PCNA^ΔSL47^-expressing cells were more sensitive to both H_2_O_2_ treatment and UV-irradiation than PCNA^WT^-expressing cells (Fig 4J and 4K). Taken together, our data suggest that PCNA Ser46 and Leu47 are important for maintaining genomic stability in cells.

### PCNA Ser46-Leu47 and ^41^DSSHV^45^ residues are required for maintaining genomic stability in an epistatic manner

**Fig 5:**
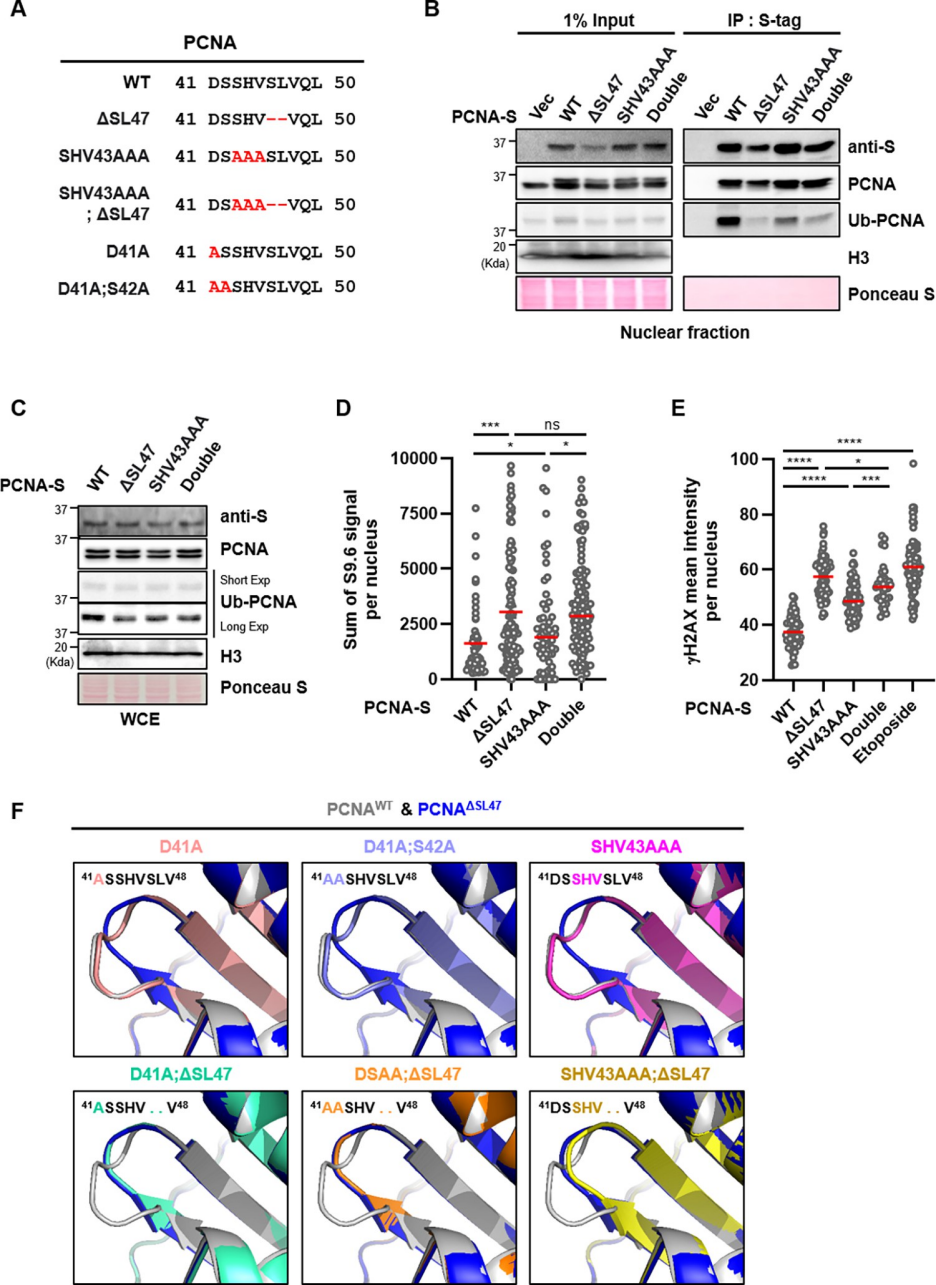
PCNA Ser46-Leu47 and ^41^DSSHV^45^ residues are required for maintaining genomic stability in an epistatic manner. (A) DNA sequences of PCNA^WT^ and central loop-associated PCNA mutants were aligned. (B) After transfection as indicated in HEK293T for 48 h, nuclear extracts were prepared for immunoprecipitation with an anti-S protein affinity bead for 2 h. Immunoprecipitated proteins were eluted, resolved by SDS-PAGE, and immunoblotted using the indicated antibodies. (C) HEK293T cells were transfected as indicated for 48 h, and whole-cell extracts were prepared for immunoblotting. (D, E) The sum intensity of S9.6 foci (D) or the mean intensity of γH2AX staining (E) was quantified and plotted. (E) 10 μM etoposide was treated for 5 h as a positive control. (F) Predicted monomer structures of human PCNA-WT, ΔSL47, D41A, D41A;S42A, and SHV43AAA and double mutant with ΔSL47 as indicated. The magnified region indicates the PCNA central loop. (D, E) Three independent experiments were performed, and one representative result is displayed. The red bar indicates the mean value. Statistical analysis: two-tailed unpaired Student’s *t*-test, **** *p* < 0.0001, *** p < 0.001, * *p* < 0.05, and n. s. = not significant.

Since PCNA central loop is encoded by ^41^DSSHV^45^ amino acids [24] and proximal to Ser46-Leu47 residues, we characterized the dependency of PCNA^ΔSL47^ on a PCNA^SHV43AAA^ mutant. Therefore, we substituted human PCNA Ser43-His44-Val45 residues into alanine (A) and generated PCNA^SHV43AAA^ as previously reported [17], and we also generated PCNA^SHV43AAA^ and PCNA^ΔSL47^ double mutant (PCNA^SHV43AAA;ΔSL47^) (Fig 5A). With these mutants, we analyzed the interaction ability of PCNA^SHV43AAA^ and PCNA^SHV43AAA;ΔSL47^ with Ub-PCNA by immunoprecipitation with S-protein affinity beads. As a result, the PCNA^SHV43AAA^ mutant showed a mild defect in PCNA ubiquitination (Fig 5B). Consistent with our observation that PCNA^ΔSL47^ impaired PCNA ubiquitination (Figs 3C, 3D and 4B–4D), we observed both PCNA^ΔSL47^ and PCNA^SHV43AAA;ΔSL47^ mutants were defective in interaction with Ub-PCNA. However, we did not observe the synergistic and additional effect on PCNA ubiquitination between PCNA^ΔSL47^ and PCNA^SHV43AAA;ΔSL47^ mutants (Fig 5B). We suspect that PCNA^ΔSL47^ mutants show a more severe phenotype than the PCNA^SHV43AAA^ mutant presumably due to the amino-acids (Ser46-Leu47) deletion, rather than alanine substitution. Ub-PCNA levels were further characterized with each mutant by immunoblotting with whole-cell lysate. As a result, cells expressing PCNA^ΔSL47^, PCNA^SHV43AAA^, and PCNA^SHV43AAA;ΔSL47^ mutants impaired PCNA ubiquitination, without synergistic effect in PCNA^SHV43AAA;ΔSL47^ mutant-expressing cells (Fig 5C).

We also investigated the DNA-RNA hybrids levels and γH2AX levels with each mutant-expressing cells by performing immunostaining. As a result, we observed that PCNA^SHV43AAA^ mutant-expressing cells showed a higher level of DNA-RNA hybrids as well as γH2AX levels compared to PCNA^WT^-expressing cells (Fig 5D and 5E). However, consistent with the effects on PCNA ubiquitination, we could not observe synergistic effects with PCNA^ΔSL47^ mutant in both DNA-RNA hybrids and γH2AX level (Fig 5D and 5E). Even in this case, PCNA^ΔSL47^ mutant-expressing cells showed a more severe phenotype than PCNA^SHV43AAA^ mutant-expressing cells.

Since the structural prediction of PCNA^ΔSL47^ showed central loop distortion (Fig 1E), and PCNA central loop is encoded by ^41^DSSH^44^ amino acids [24], we predicted PCNA^D41A^, PCNA^D41A;S42A^, and PCNA^SHV43AAA^ monomer structures by using Alphafold2. However, we could not find PCNA central loop distortion in PCNA^D41A^, PCNA^D41A;S42A^, and PCNA^SHV43AAA^ by structure prediction (Fig 5F). Interestingly, when we predicted those alanine-substituted PCNA mutants with PCNA^ΔSL47^ double mutants (PCNA^D41A;ΔSL47^, PCNA^DSAA;ΔSL47^, and PCNA^SHV43AAA;ΔSL47^), we observed central loop distortion (Fig 5F). Taken together, these data suggest that PCNA^D41A^, PCNA^D41A;S42A^, and PCNA^SHV43AAA^ mutants are dependent on the PCNA^ΔSL47^ mutant in inducing PCNA central loop distortion. Collectively, we concluded that PCNA Ser46-Leu47 and ^41^DSSHV^45^ residues are required in an epistatic manner for maintaining genomic stability.

## Discussion

**Fig 6:**
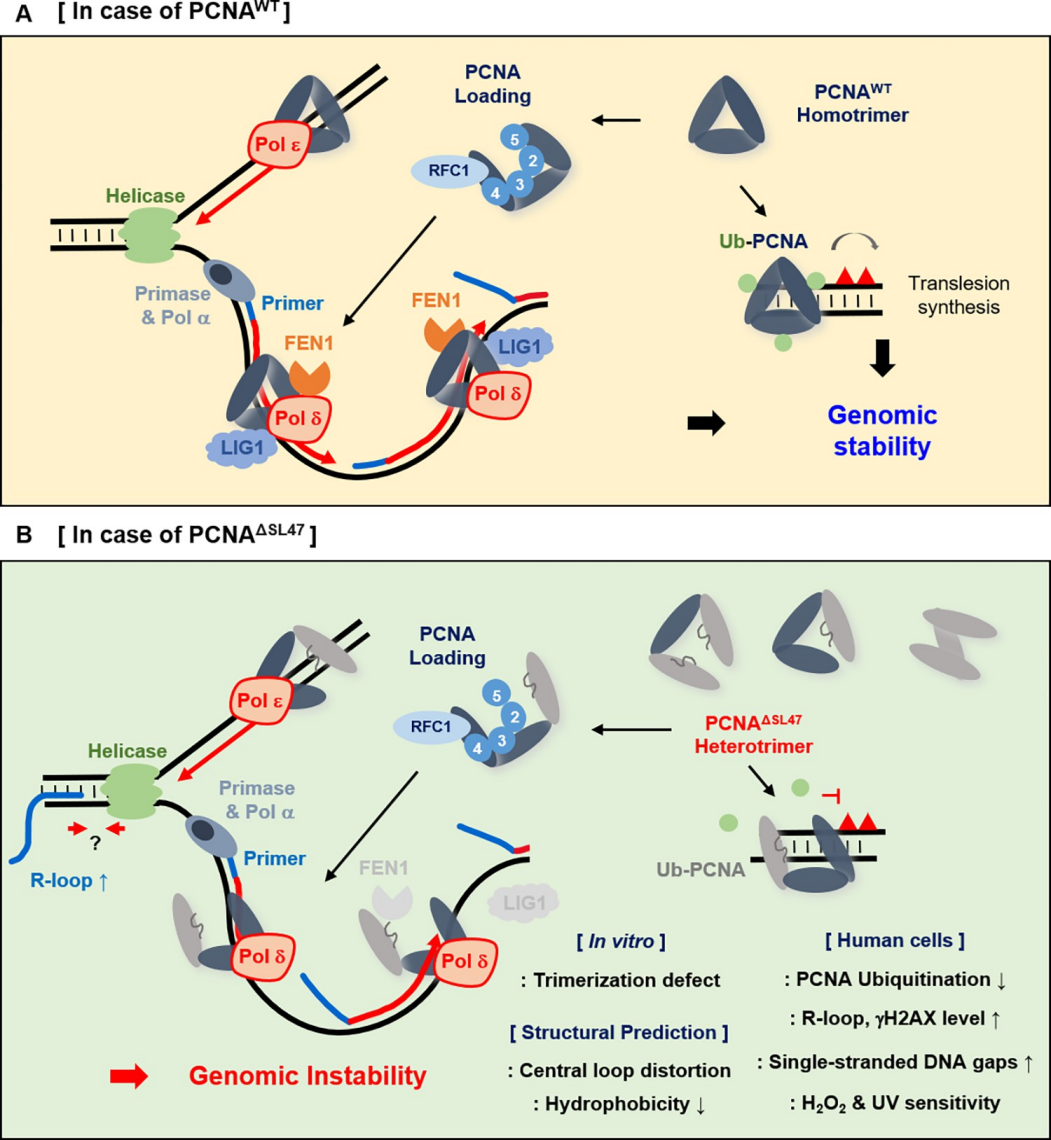
Graphical models of PCNA Ser46 and Leu47 residues are crucial in genomic integrity. (A) During DNA replication, PCNA^WT^ forms a homotrimer and is loaded onto the lagging strand of DNA by the RFC. After RNA primer synthesis by primase and Pol α at the lagging strand, PCNA and POL δ-meditated DNA synthesis occurs. FEN1 and LIG1 interact with PCNA^WT^ homotrimer and process 5’ overhang flap structures and seal the nick. In addition, when the DNA replication fork encounters DNA lesions, PCNA is ubiquitinated and proceeds with translesion synthesis (TLS). Therefore, normal DNA replication proceeds and ensures genomic integrity. (B) When Ser46-Leu47 residues of PCNA are deleted, the PCNA^ΔSL47^ mutation monomer disrupts the PCNA trimerization *in vitro*. The central loop formation of the PCNA^ΔSL47^ mutant is predicted to be structurally distorted and its hydrophobicity is expected to decrease. PCNA ubiquitination is defective, and high levels of DNA-RNA hybrids and γH2AX are detected in PCNA^ΔSL47^-expressing cells. Consistently, PCNA^ΔSL47^-expressing cells are sensitive to several DNA-damaging agents. Taken together, PCNA Ser46-Leu47 residues are crucial for genomic integrity.

PCNA forms a homotrimer and plays a crucial role in DNA metabolism, including DNA replication and repair. Therefore, mutations of PCNA have severe effects on the maintenance of genomic integrity. In this study, we identify the PCNA Ser46-Leu47 residues that are crucial in maintaining genomic integrity (Fig 6).

Although there are several differences among the PCNA central loop-associated mutants (D41A, D41A;S42A, SHV43AAA, and ΔSL47), these mutants do not show synergistic effects in PCNA ubiquitination, regulation of DNA-RNA hybrids and γH2AX levels, and putative central loop distortion (Fig 5). Previous studies have shown that PCNA D41 residue is required for RFC, FEN1, and Pol δ binding, while PCNA trimerization is intact [26,27,45–47]. In the case of PCNA^SHV43AAA^ mutant, it impairs DNA loading by RFC and FEN1 stimulation, while PCNA trimerization is intact [17]. In our PCNA^ΔSL47^ mutant, we observed defects in PCNA trimerization, *in vitro* loading, FEN1 and LIG1 binding, central loop formation, and PCNA ubiquitination, while chromatin loading is intact probably due to the presence of PCNA^WT^ and its compensation. We suspect that the observed differences among these PCNA mutants are likely caused by the alanine substitution or deletion mutant. Furthermore, it implies that the central loop of PCNA, which forms a hydrophobic core and enables interactions with various proteins, is intricately regulated.

FEN1 and LIG1 interact with PCNA through its PCNA-interacting protein-box (PIP-box) motif, and their processing and ligase activities are crucial for Okazaki fragment maturation (OFM) [1,48,49]. Our results suggest that the PCNA^ΔSL47^ mutant is defective in FEN1 and/or LIG1 interaction (Fig 3C–3F), which might result in OFM failure. Furthermore, in unperturbed condition, we observed an increased in alkaline tail moment in PCNA^ΔSL47^-expressing cells (Fig 4I). This suggests that PCNA^ΔSL47^-expressing cells might fail to complete OFM, resulting in increased single-straned DNA (ssDNA) gaps. It is noteworthy that cells expressing PCNA K164R mutant and RAD18 knockout cells, which are defective in PCNA ubiquitination, also exhibit OFM failure [19]. Notably, the PCNA S228I mutation impairs FEN1 and LIG1 interactions and results in clinical features, including short stature, hearing loss, premature aging, telangiectasia, neurodegeneration, and photosensitivity in humans [30].

After DNA synthesis or damage bypass, PCNA and/or Ub-PCNA are unloaded by the ATAD5-RFC-like complex (RLC) [7–9]. Therefore, in ATAD5-deficient cells, unprocessed PCNA on the DNA causes defects in replication fork restart under replication stress, defects in single-strand break repair, and generates harmful R-loop formation by conflicting with transcription machinery [39,50,51]. Since ATAD5-RLC unloads PCNA after DNA synthesis, abnormal PCNA^ΔSL47^ protein adducts cannot be unloaded after replication fork passage in ATAD5-deficient cells. However, at present, it is unknown whether ATAD5-RLC can unload PCNA^ΔSL47^ protein adducts even though PCNA^ΔSL47^ can interact with RFC subunits. We suspect that PCNA^ΔSL47^ may show a severe phenotype in ATAD5-deficient cells, and further studies are required to confirm this.

According to *in vitro* experimental results, PCNA should be loaded onto chromatin for its ubiquitination, since soluble PCNA which is not loaded onto DNA cannot be ubiquitinated [7,38]. Consistent with our previous report, PCNA^ΔSL47^ mutants are loaded onto DNA to a similar extent to PCNA^WT^ [31], but chromatin-bound Ub-PCNA level is significantly reduced (Fig 4D). Therefore, we suspect at least PCNA SUMOylation, which occurs at the same residue of PCNA (K164) and has superimposed but distinct structures [52,53], may have defects similar to PCNA ubiquitination in PCNA^ΔSL47^-expressing cells. Not only SUMOylation, but also other PCNA post-translational modifications (PTMs) such as acetylation (K13, K14, K77, K80), methylation (K110, K248), and phosphorylation (Y211) and their role have been characterized [11,54–57]. Although we cannot expect the effects of PCNA^ΔSL47^ on PCNA PTMs in addition to its ubiquitination, further studies are required to characterize the remained mechanisms.

We observed that abolished PCNA^ΔSL47^ loading onto DNA by RFC *in vitro* PCNA loading assay, while interaction between PCNA^ΔSL47^ and RFC1 or RFC4 is intact in cells (Figs 2C, 3A and 3B). There are two major differences between the *in vitro* PCNA loading assay and PCNA loading in cells by RFC. 1) First difference is about the presence or absence of PCNA^WT^ monomers. In the *in vitro* assay, only PCNA^WT^ or PCNA^ΔSL47^ proteins are present respectively. Therefore, PCNA^ΔSL47^ monomers form a trimer by themselves, without the presence of PCNA^WT^ monomers. However, in cells, there are basal levels of endogenous PCNA^WT^ monomers. 2) The other difference is about the usage of N-terminal deleted RFC1^ΔN554^ purified protein for *in vitro* PCNA loading assay. We purified RFC1^ΔN554^ protein, contains RFC1 C-terminus from 555–1148 and is reported to form the RFC complex with enhanced activity [58], for an *in vitro* PCNA loading reaction (Fig 2A). In particular, it is important to consider the complexity of the binding mode between RFC and the PCNA homotrimer, as well as the distinct mechanisms for PCNA recognition and loading by RFC [12,14,59]. The interaction between the hRFC1 and PCNA is the most extensive, primarily through hydrophobic interactions [14]. And the RFC1 N-terminal BRCT domain promotes and is required for PCNA loading [12]. Therefore, although RFC1, RFC4, and PCNA^ΔSL47^ interaction is intact in cells (Fig 3A and 3B), we are not sure whether PCNA^ΔSL47^ protein can interact with RFC1^ΔN554^ *in vitro*. In addition, it is difficult to predict the interactions between the other RFC subunits (RFC2, RFC3, RFC5) that we have not tested, or the structure during docking with PCNA^ΔSL47^, in the absence of PCNA^WT^ monomer *in vitro*. Importantly, the deletion of additional N-terminal amino acids of RFC1 (RFC1^ΔN604^) abolishes PCNA loading while PCNA interaction is intact [12], suggesting PCNA loading and PCNA recognition can be distinct. Therefore, we suspect that the interaction between PCNA and RFC1 observed in cells may not be essential for PCNA loading, especially PCNA^ΔSL47^ *in vitro*. Further studies are required to determine how RFC opens and interacts with PCNA^ΔSL47^ *in vitro*.

## Materials and methods

### Cell lines and cell culture

HEK293T and HeLa cells were cultured in DMEM (HyClone) supplemented with 10% fetal bovine serum (HyClone), 100 U/mL penicillin G (Life Technologies), and 100 mg/mL streptomycin (Life Technologies) in a humidified atmosphere of 5% CO_2_ at 37°C. Plasmids expressing HA-tagged PCNA constructs were cloned into the pcDNA5 FRT-TO, and S-tagged PCNA constructs were cloned into the pcDNA3 mammalian expression vector. The V5(GKPIPNPLLGLDST) epitope-tagged FEN1 and LIG1 constructs were cloned into a pcDNA5 FRT-TO mammalian expression vector. Transfection of plasmid DNA was performed using X-tremeGENE™ HP (#6366546001, Roche) or Transporter 5 (#26008–5, Polysciences) and siRNAs were transfected using RNAiMAX (#13778500, Thermo Fisher). Cells were analyzed 48 h after transfection.

### siRNAs

Synthetic duplex small interfering RNA (siRNA) was purchased from Bioneer and used as control siRNA (Bioneer #SN-1002). PCNA 3’-untranslated region (UTR)-targeting siRNA was purchased from Dharmacon^TM^ (#A-003289-25).

### Chemicals, reagents, and antibodies

The following chemicals were used in this study: ML323 (S7529, an USP1-UAF1 inhibitor, 30 μM) (Selleckchem), and Dynabeads M-280 streptavidin (11206D) (Invitrogen). The following reagents were used in this study: Ultrapure 20X SSC (#15557044) (Invitrogen), hydrogen peroxide solution (H_2_O_2_) (H1009) (Sigma-Aldrich), formaldehyde (P2031), and formamide (FC1014) (Biosesang). The following antibodies were used: anti-PCNA (ab18197), anti-LIG1 (ab615) (Abcam), anti-ubiquityl-PCNA (Ub-PCNA) (Lys164) (13439S), anti-histone H3 (9175), anti-CHK1 (2360), anti-pCHK1 (S345) (2348), anti-γH2AX (9718) for immunoblotting (Cell signaling), anti-FLAG (F3165), anti-HA (H9658), anti-V5 (V8137), anti-Myc (05–724), anti-S tag (SAB2702227), α-tubulin (T9026) (Sigma-Aldrich), anti-RFC1 (NBP2-54960) (Novus Biologicals), anti-FEN1 (sc-13051) (Santa Cruz), anti-RPA32 (A300-244A), anti-pRPA32 (S4/S8) (A300-245A), anti-pRPA32 (S33) (A300-246A) (Bethyl), anti-S9.6 (ENH001) (Kerafast), and anti-γH2AX (05–036) for immunostaining (Millipore).

### Protein purification

His-tagged Human PCNA wild-type (PCNA^WT^), ΔSL^47^ (PCNA^ΔSL47^), and FLAG-tagged TALE proteins were expressed in BL21 cells (#EC0114, Thermo Fisher). RFC (RFC1^ΔN554^, and RFC2-5) were purified using the Bac-to-Bac Baculovirus expression system (#10359016, Thermo Fisher) as previously described [7]. We used the RFC1^ΔN554^ protein because it is sufficient for in vitro PCNA loading assays [58]. Viruses were prepared using Sf9 cells (#11496015, Thermo Fisher Scientific), and proteins were expressed in Hi-5 cells (#B85502, Thermo Fisher Scientific). To purify proteins from E. coli, 2 L cultures were grown to an optical density of 0.4, and protein expression was induced by the addition of 0.5 mM IPTG for 6 h at 37°C. Cells were harvested and resuspended in by lysis buffer 300 mM KCl Buffer H (25 mM HEPES at pH 7.5, 1 mM EDTA, 1 mM EGTA, 2.5 mM magnesium acetate, 10% glycerol, 1 mM DTT, 0.02% NP40, with 1x complete protease inhibitor cocktail (Roche)). PCNA lysates were clarified by ultracentrifugation (36,000 rcf, 60 min), and proteins were purified by sequential application of a complete His-tag resin and ion exchange chromatography. TALE lysates were purified by sequential application of complete His-tag resin, anti-FLAG M2 agarose resin, and ion exchange chromatography. The purified proteins were analyzed by SDS-PAGE and Coomassie blue staining. Aliquoted proteins were frozen and stored at −80°C.

### DNA substrates for PCNA loading reaction

A 130-mer DNA substrate was prepared by annealing two oligonucleotides to 130-mer oligonucleotides (CTCTA TAAGA TATAG TCAAG TTCAG ACGTC CATGC CCTTA TCGGA GTCTC CGGCA AATGC AATGC TCAGC ATCTC CAGCC GCTTA GCATA CTTGC CGATG TACAT GAAAG CTTTG TCTGT CAGCA GGCCG). One oligonucleotide was a 5′ biotinylated 50-mer (CGGCC TGCTG ACAGA CAAAG CTTTC ATGTA CATCG GCAAG TATGC TAAGC) annealed to the 3′ portion of 130-mer oligonucleotides, and the other was a 70-mer (GCTGA GCATT GCATT TGCCG GAGAC TCCGA TAAGG GCATG GACGT CTGAA CTTGA CTATA TCTTA TAGAG) annealed to the 5′ portion of the 130-mer. The resulting DNA substrates contained a 10 nucleotides gap. The annealed primer-template DNA was attached to streptavidin-coated magnetic beads (Dynabeads M-280, Invitrogen) and unbound DNA/oligonucleotides were washed out. Bead-attached DNA was resuspended in 300 mM potassium acetate KoAc Buffer H without magnesium acetate. Each substrate had a TALE-binding sequence on the opposite side of the biotinylation. 0.5 pmol DNA substrate was pre-incubated with 50 nM TALE in 40 μL reaction mixture (25 mM HEPES pH 7.5, 300 mM KoAc, 1 mM EDTA, 1 mM EGTA, 2.5 mM magnesium acetate, 10% glycerol, 1 mM DTT, 1 mM ATP, 0.02% NP40) at 37°C for 30 min before the PCNA loading reaction.

### *In vitro* PCNA loading reaction

The PCNA loading reaction was performed as previously described [7]. Two buffers were used for the PCNA-loading reaction. A standard 2× loading reaction buffer (50 mM HEPES [pH 7.5]), 24 mM magnesium acetate, 0.2 mM zinc acetate, 2 mM dithiothreitol [DTT], 40 mM phosphocreatine, 12 mM ATP, 0.04% NP40, 20% glycerol, 0.8 mg/mL bovine serum albumin, and 2× complete protease inhibitor cocktail [Roche]) and a protein dilution buffer (25 mM HEPES [pH 7.5], 300 mM KoAc, 1 mM EDTA, 1 mM EGTA, 2.5 mM magnesium acetate, 10% glycerol, 1 mM DTT, 1 mM ATP, and 0.02% NP40). Initially, TALE protein was incubated with DNA substrate in a thermomixer (Eppendorf) for 30 minutes at 37°C and 1200 rpm. After the incubation, TALE-bound beads were washed with 0.5 M KCl, 1 M KCl, and 300 mM KCl Buffer H (25 mM HEPES [pH 7.5], 1 mM EDTA, 1 mM EGTA, 2.5 mM magnesium acetate (MgoAc), 10% glycerol) sequentially. Then, 20 μL of 2× loading reaction buffer was mixed with 20 μL of protein mix containing RFC (5 nM or the indicated amount) and PCNA (500 nM). The PCNA-loading reaction was performed by adding 40 μL of the reaction mixture to the bead-conjugated DNA substrate. The reaction mixture was incubated in a thermomixer (Eppendorf) for 30 min at 37°C and 1200 rpm. After the reaction, the remaining RFC and unbound PCNA were removed by washing the beads once with 0.3 M KCl, once with 0.5 M KCl, and once again with 0.3 M KCl Buffer H. DNA was digested for 10 min, beads were collected using a magnet, and supernatants were taken. PCNA levels in the DNA were analyzed by immunoblotting.

### Protein extraction and immunoblot analysis

Protein extraction and immunoblot analyses were performed as previously described [39]. For whole-cell proteins extraction, cell pellets were lysed in RIPA buffer (50 mM Tris-HCl, pH 8.0, 150 mM NaCl, 5 mM EDTA, 1% Triton X-100, 0.1% sodium dodecyl sulfate, 0.5% sodium deoxycholate, 0.1 M phenylmethylsulfonyl fluoride (PMSF), phosphatase inhibitors, and protease inhibitors) with Benzonase nuclease for 45 min at 4°C, followed by sonication and centrifugation. For chromatin fractionation, cell pellets were resuspended in Buffer A (100 mM NaCl, 300 mM Sucrose, 3 mM MgCl_2_, 10 mM PIPES, pH 6.8, 1 mM EGTA, 0.2% Triton X-100, 0.1 M PMSF, phosphatase inhibitors and protease inhibitors) and incubated for 10 min at 4°C. After centrifugation, pellets were further lysed in RIPA buffer with Benzonase nuclease for 45 min at 4°C. For immunoblot analysis, the proteins were separated by sodium dodecyl sulfate-polyacrylamide gel electrophoresis and transferred to a nitrocellulose membrane. The blot was blocked with Tris-buffered saline containing 0.1% Tween 20 (TBST) supplemented with 5% skim milk for 30 min and incubated with primary antibodies overnight. After washing with TBST buffer, the blot was incubated with HRP-conjugated secondary antibodies (Enzo Life Sciences) for 30 min. After washing with TBST buffer, the signal was detected using an enhanced chemiluminescence reagent (Thermo Fisher Scientific) and an automated imaging system (ChemiDoc™; Bio-Rad Laboratories).

### Immunoprecipitation (IP)

Isolation of a nuclear protein fraction and a Triton X-100–insoluble fraction (chromatin-bound fraction), immunoprecipitation, and immunoblot analyses were performed as previously described, with slight modifications [39]. For nuclear protein extraction, cell pellets were resuspended in MBS-A buffer (10 mM HEPES (pH 7.5), 340 mM sucrose, 1.5 mM MgCl_2_, 10 mM KCl, 10%glycerol, 1 mM DTT, 0.1 M PMSF, phosphatase inhibitors and protease inhibitors) and incubated for 8 min at 4°C. After centrifugation, nuclei were further lysed in buffer X (100 mM Tris–HCl, 250 mM NaCl, 1mM EDTA, 1% NP-40, 0.1 M PMSF, phosphatase inhibitors, and protease inhibitors) with Benzonase nuclease followed by sonication and centrifugation. Anti-FLAG M2 agarose (A2220, Sigma), anti-c-Myc agarose (A7470, Sigma), anti-HA agarose (A2095, Sigma), anti-V5 agarose (A7345, Sigma), and anti-S-protein agarose (69704, Novagen) affinity beads were used for immunoprecipitation, as described previously. Agarose beads were incubated with cell lysates for 2 h at 4°C, washed three times with Buffer X at 4°C, and bound proteins were eluted with FLAG-peptide (19–73301, Peptron) or collected by denaturation with 2X SDS loading buffer and resolved by SDS-PAGE and immunoblotting.

### Cell survival assay

The cell survival assay was performed as previously described [50]. Briefly, cells were seeded in 96-well culture plates. After 24 h, cells were treated with drugs, and cell survival was measured by the MTT assay or by using the CellTiter-Glo® 2.0 Assay Kit (G7572, Promega) according to the manufacturer’s instructions. For the MTT assay, MTT reagent was added together with a different range of H_2_O_2_ for 4 h, the purple formazan crystal was dissolved with DMSO, and the colored solution was quantified using a plate reader. For the CellTiter-Glo® 2.0 Assay, cells were lysed with CellTiter-Glo® reagent and the luminescence signal was read using a plate reader.

### Alkaline COMET assay (Single-cell gel electrophoresis)

The COMET assay was performed as previously described [50]. In brief, each cell suspension was mixed with COMET LM Agarose at 37°C and the mixture was spread on a COMET slide (Trevigen). After solidification of the agarose, the slide was immersed in a lysis solution (Trevigen) for 1 h at 4°C. Images were acquired with a fluorescence microscope (BX53; Olympus, Tokyo, Japan), and the tail moment was calculated using CometScore software version 2.0.

### Immunostaining and image acquisition

Cells plated on LabTek™ chamber slides (Thermo Fisher Scientific) were fixed and stained as previously described [50] with slight modifications. Briefly, the cells were pre-extracted with CSK buffer for 10 min on ice and fixed with 4% paraformaldehyde (PFA) for 20 min at room temperature. Fixed cells were washed with phosphate-buffered saline (PBS), incubated in blocking buffer (10% FBS in PBS) for 30 min, and then incubated with the indicated antibodies diluted in blocking buffer at 4°C overnight. After three washes with 0.05% Triton X-100 in PBS, Alexa Fluor®-conjugated secondary antibodies (Thermo Fisher Scientific) were added and incubated for 30 min. After washing, the cells were mounted using the ProLong®Gold antifade reagent (Vector Laboratories, Burlingame, CA, USA). Confocal images were acquired using an LSM880 confocal microscope (Carl Zeiss) with a Z-stack process and 40×/1.2 lens objective. Image acquisition and analysis were performed using Zen 2.6 (blue edition) (Carl Zeiss) software.

### Site-directed mutagenesis

Site-directed mutagenesis was performed using the QuikChange site-directed mutagenesis kit (#210518, Agilent Technologies) according to the manufacturer’s instructions to generate plasmid DNA for the PCNA^ΔSL47^, PCNA^SHV43AAA^, and PCNA^SHV43AAA;ΔSL47^ mutants. The constructs were confirmed by sequencing.

### Protein structure prediction using AlphaFold2

The PCNA amino acid sequences were downloaded from the UniProt database (https://www.uniprot.org/, accessed on March 27, 2022). AlphaFold2 was downloaded from GitHub and installed as previously described (https://github.com/deepmind/alphafold, accessed February 3, 2022). Protein structure prediction was performed using recommended codes [60,61]. Pymol 1.4.1 (https://www.pymol.org/, accessed June 25, 2022) was used to analyze the predicted protein structure.

### Statistical analysis

Prism 9 (GraphPad Software) was used to generate graphs and analyze the data. Statistical analyses were performed using a two-tailed unpaired Student’s *t*-test. All experiments were performed at least three times, and the graphs show the average of three independent experiments. Error bars indicate standard error or mean (s.e.m.); **** *p* < 0.0001, *** *p* < 0.001, ** *p* <0.01, * *p* < 0.05, and n. s. = not significant.

## Supporting information

S1 Fig The structural importance of PCNA Ser46-Leu47 residues in forming a central loop in zebrafish, related to Fig 1. (A-C) Predicted trimerized PCNA structure analyzed with WT-WT-WT, WT-WT-ΔSL47, WT-ΔSL47-ΔSL47, ΔSL47-ΔSL47 combinations in zebrafish. PCNA WT-WT-WT homotrimer (Gray) are structurally aligned with either WT-WT-ΔSL47 (Green) (A), WT-ΔSL47-ΔSL47 (Cyan) (B), ΔSL47-ΔSL47-ΔSL47 (Yellow) (C). Either the front or side view is displayed (Top). The central loop regions are colored as indicated (Bottom).
(TIF)

S1 File (XLSX)

S1 Raw images Original uncropped underlying all blot or gel results (Corresponding to Fig 3). (TIF)

S2 Raw images Original uncropped underlying all blot or gel results (Corresponding to Fig 2). (TIF)

S3 Raw images Original uncropped underlying all blot or gel results (Corresponding to Fig 3). (TIF)

S4 Raw images (TIF)

S5 Raw images Original uncropped underlying all blot or gel results (Corresponding to Fig 5). (TIF)
