# Supplementary figures and images for: PCNA Ser46-Leu47 residues are crucial in preserving genomic integrity
Source: PLoS One. 2023 May 19;18(5):e0285337. doi: 10.1371/journal.pone.0285337 (PMC10198555; doi:10.1371/journal.pone.0285337)

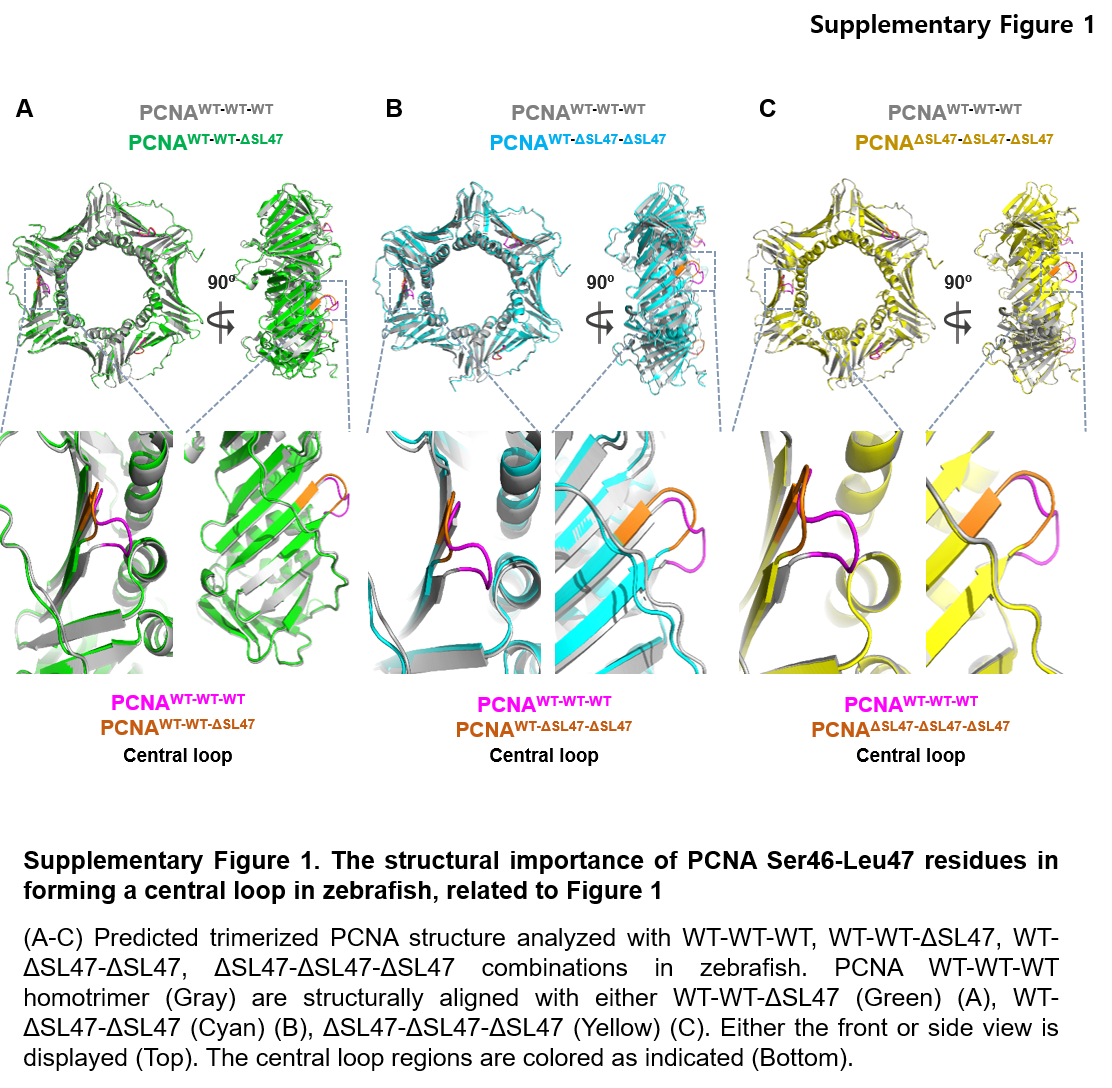

Supplement: S1 Fig — (A-C) Predicted trimerized PCNA structure analyzed with WT-WT-WT, WT-WT-ΔSL47, WT-ΔSL47-ΔSL47, ΔSL47-ΔSL47 combinations in zebrafish. PCNA WT-WT-WT homotrimer (Gray) are structurally aligned with either WT-WT-ΔSL47 (Green) (A), WT-ΔSL47-ΔSL47 (Cyan) (B), ΔSL47-ΔSL47-ΔSL47 (Yellow) (C). Either the front or side view is displayed (Top). The central loop regions are colored as indicated (Bottom). (TIF) [file pone.0285337.s001.tif]

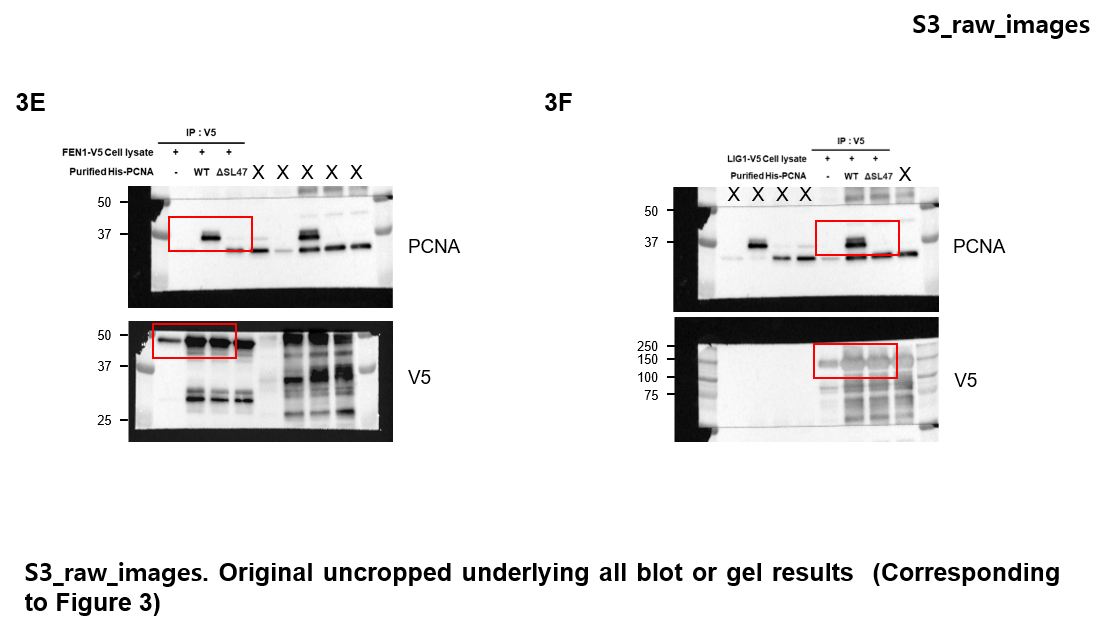

Supplement: S1 Raw images — (TIF) [file pone.0285337.s003.tif]

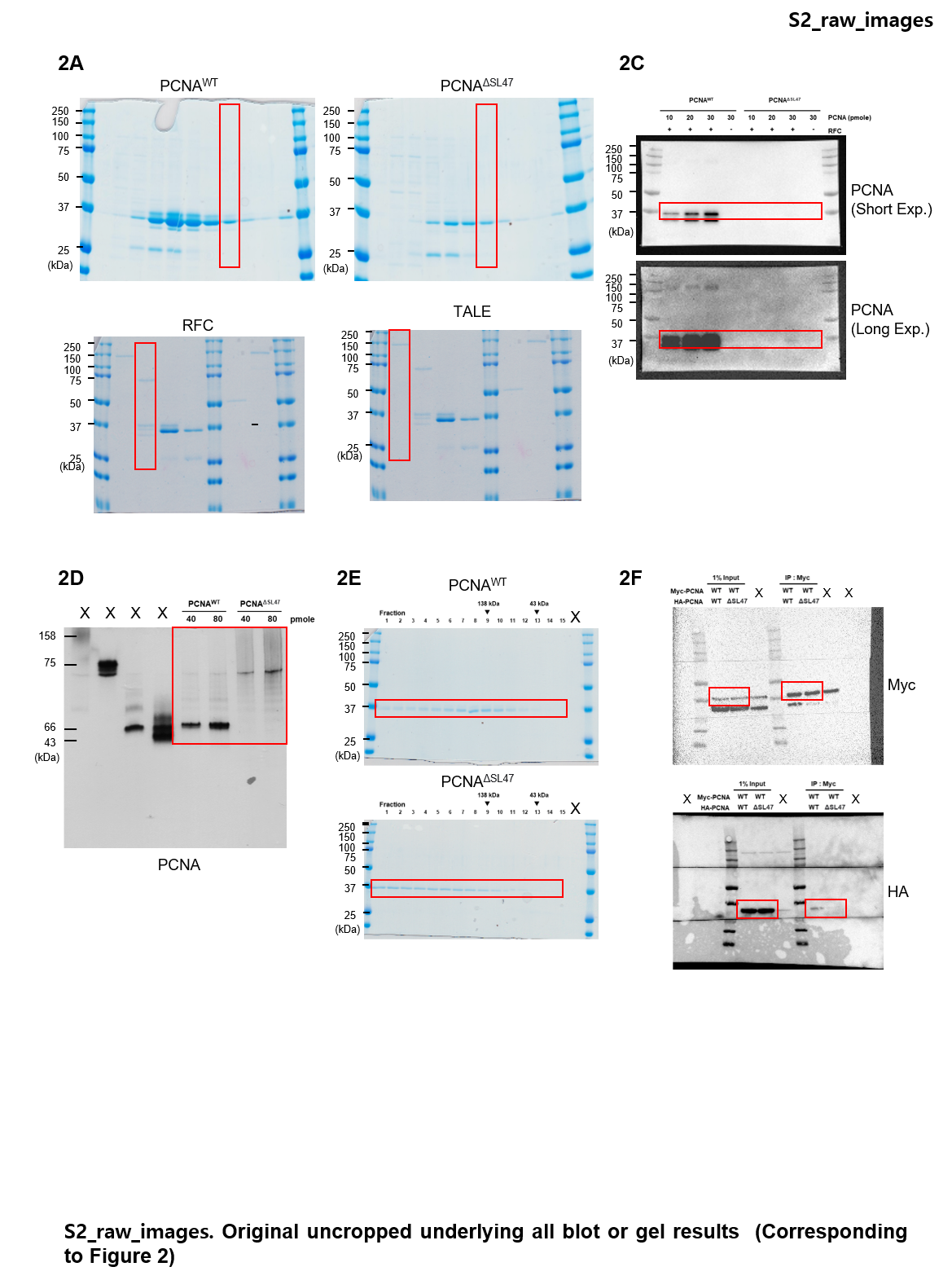

Supplement: S2 Raw images — (TIF) [file pone.0285337.s004.tif]

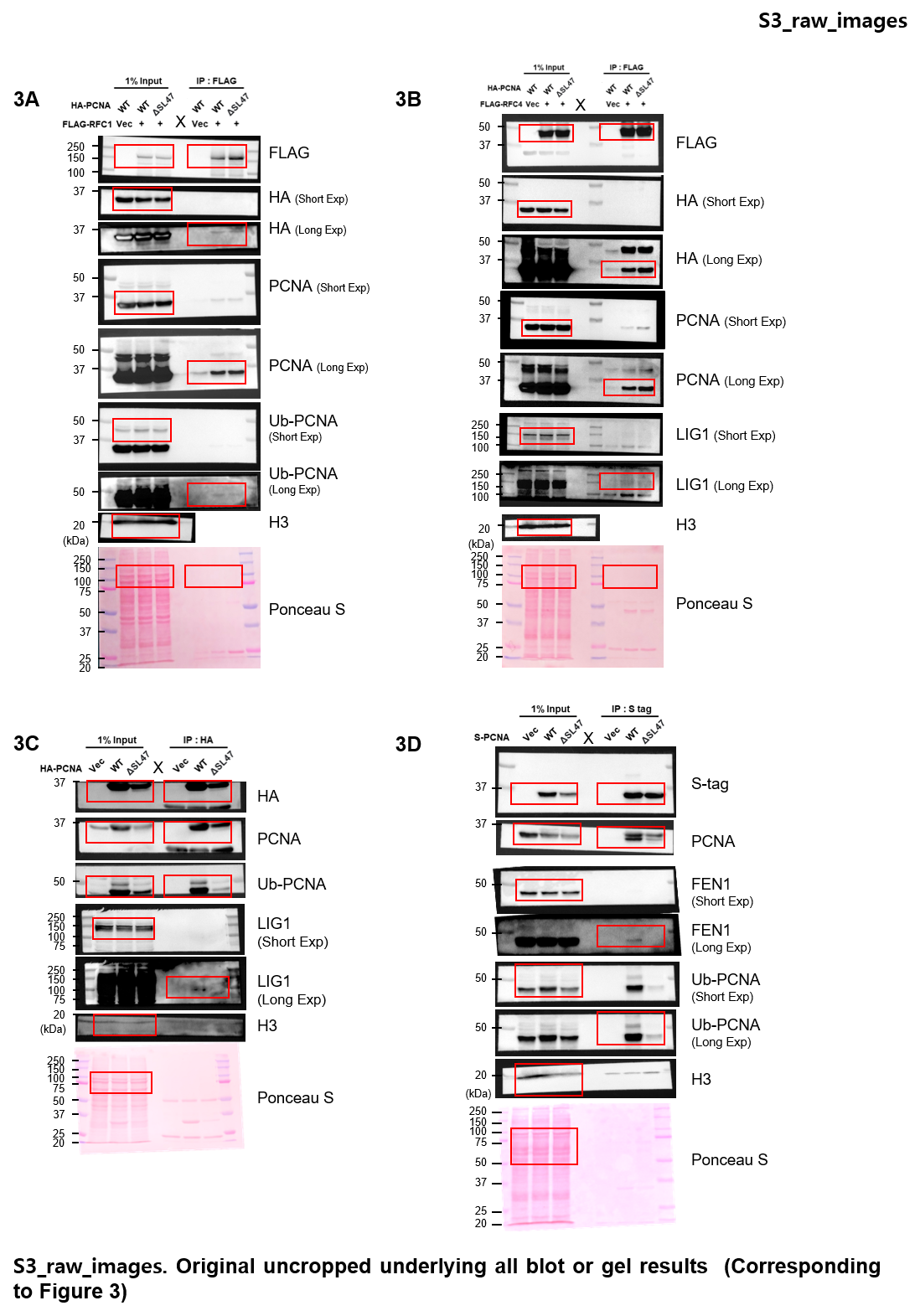

Supplement: S3 Raw images — (TIF) [file pone.0285337.s005.tif]

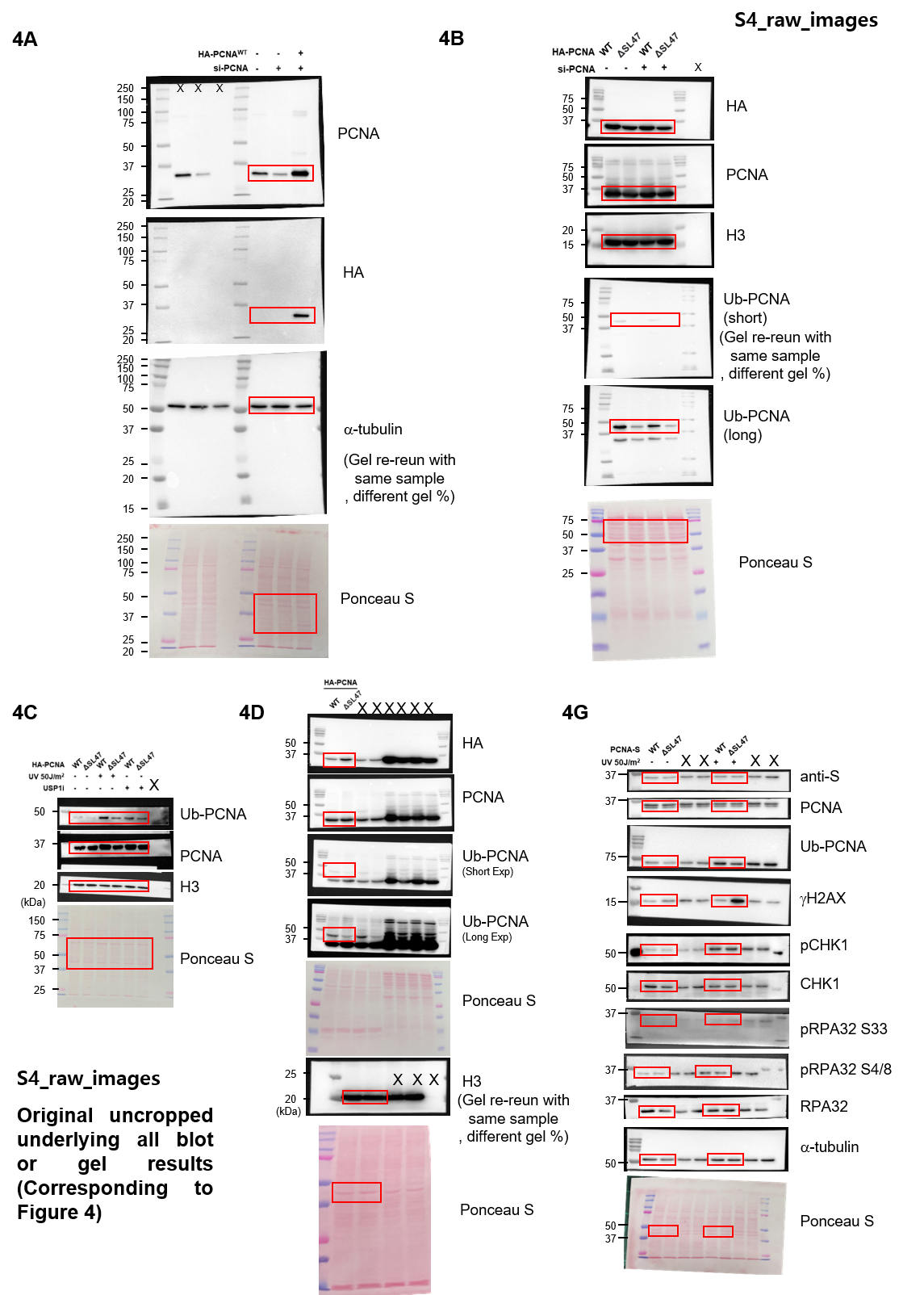

Supplement: S4 Raw images — (TIF) [file pone.0285337.s006.tif]

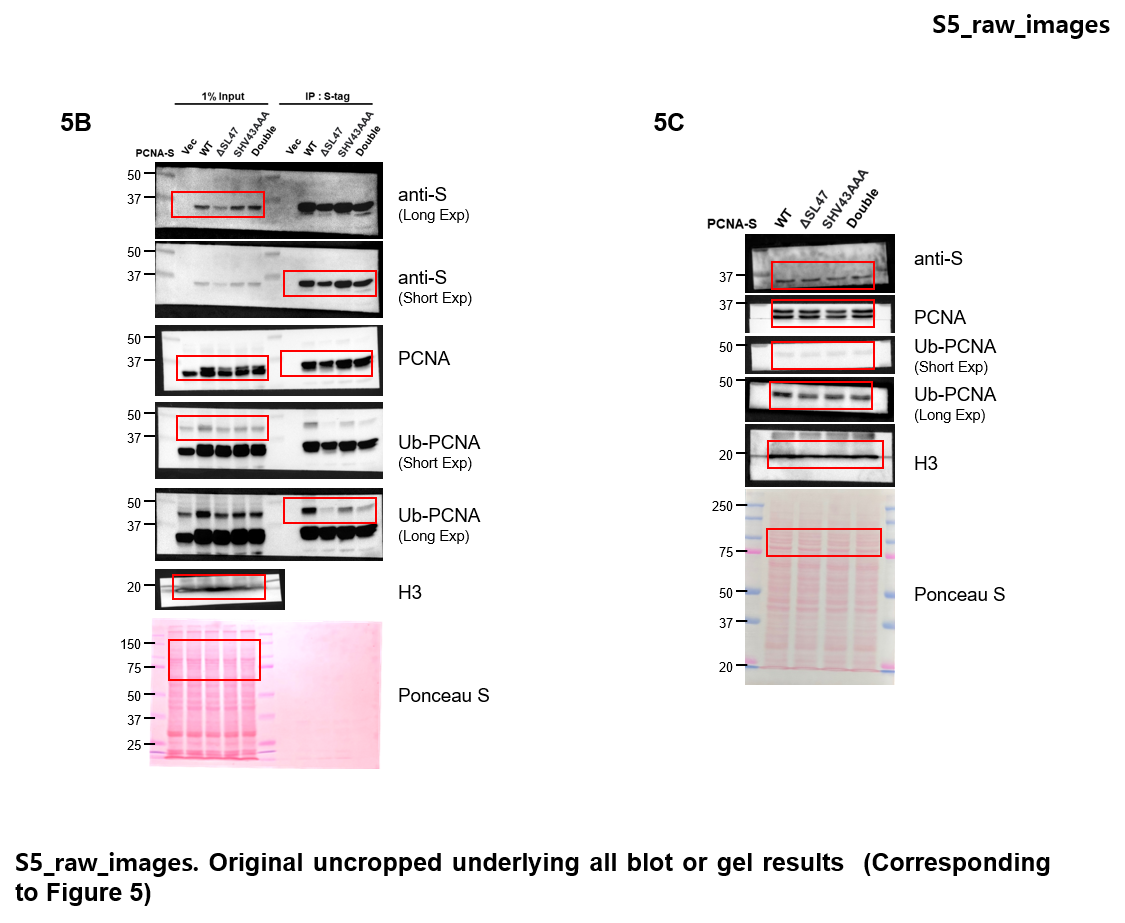

Supplement: S5 Raw images — (TIF) [file pone.0285337.s007.tif]
